# Lamarck redux and other false arguments against SARS‐CoV‐2 vaccination: Pseudoscientific arguments against vaccination do not stand up to scrutiny

**DOI:** 10.15252/embr.202254675

**Published:** 2022-02-23

**Authors:** Emanuel Goldman

**Affiliations:** ^1^ Rutgers New Jersey Medical School Newark NJ USA

**Keywords:** Economics, Law & Politics, Microbiology, Virology & Host Pathogen Interaction, Science Policy & Publishing

## Abstract

The COVID‐19 pandemic has triggered a new bout of anti‐vaccination propaganda. These are often grounded in pseudoscience and misinterpretation of evolutionary biology.

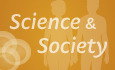

Towards the end of summer of 2021, there seemed cause for cautious optimism for putting this pandemic behind us. It was clear that the route of viral transmission was airborne and not via surfaces (Goldman, 2021a), which means that masks are very efficient at reducing the spread of SARS‐CoV‐2. The number of cases in the United States and Europe were declining, and the first vaccines became available with many people lining up to get their jabs. But not all. A significant portion of the population have been refusing to get vaccinated, some of whom were fooled or encouraged by pseudoscientific misinformation propagated on the Internet.

## Lamarck's “adaptive evolution” resurrected

As a microbiologist, I knew that the more opportunities the virus is afforded to grow, the greater the risk that new and potentially deadlier mutants could evolve. The Delta variant had already begun gaining a foothold in the population, with early reports of breakthrough infections in vaccinated people. When the *Proceedings of the National Academy of Sciences USA* published a paper describing yet another new variant from Brazil, I sent a letter to the *Proceedings* describing how those who choose to remain unvaccinated pose a threat to the vaccinated (Goldman, 2021b). After *How the unvaccinated threaten the vaccinated for COVID‐19: A Darwinian perspective* was published, I received a lot of email explaining how I was wrong, some of it pointing to a Twitter post by Geert Vanden Bossche, an independent virologist and self‐described vaccine expert, attacking my letter. Vanden Bossche has achieved some public attention for publicizing an open letter to the World Health Organization opposing vaccinations against SARS‐CoV‐2; his letter has been seized upon by advocates against vaccination as it seems to provide a veneer of scientific legitimacy to their antivaccination argument.

A significant portion of the population refuse to get vaccinated, some of whom are fooled or encouraged by pseudoscientific misinformation propagated on the Internet.

Most of Vanden Bossche’s arguments against SARS‐CoV‐2 vaccination have been disproven (https://sciencebasedmedicine.org/countering‐geert‐vanden‐bossches‐dubious‐viral‐open‐letter‐warning‐against‐mass‐covid‐19‐vaccination/), but there is one fundamental aspect of his arguments that I have not seen fully addressed. The crux of Vanden Bossche’s and his disciples’ anti‐vaccination argument is essentially a resurrection of Lamarck's theories of adaptive evolution. Jean Baptiste Lamarck (1744–1829), rightly or wrongly, is most remembered for advocating the “inheritance of acquired characteristics”, which was discredited in modern biology almost 80 years ago. With respect to SARS‐CoV‐2 and vaccination, the argument goes that when the virus infects a vaccinated person, it acquires the ability to evade the immunity conferred by the vaccine, thereby becoming capable of infecting and growing in vaccinated hosts and immune individuals who recovered from COVID‐19. From their point of view, vaccination is thus fuelling the generation of immune‐resistant variants, which could ultimately become an existential danger to humanity. Thus, they claim, it is the vaccinated who threaten the unvaccinated, the opposite of what I argued in my letter in the *Proceedings*.

## Vaccination does not *generate* variants resistant to the vaccine

What is wrong with this argument is that modern biology has demonstrated that mutation is random, not directed by the environment. The classic fluctuation test experiments of Salvador Edward Luria and Max Delbrück, published in 1943, showed that a bacterial cell's resistance to a virus is not induced by the virus, but results from random mutations that allow the cell to resist the virus. In other words, mutations occur without selective pressure, not the other way around. Thus, the evolution and inheritance of new characteristics is not subject to Lamarckism even if epigenetics and hypermutation provide some important modifiers to the underlying mutation‐selection process.

Luria and Delbruck, who were awarded the Nobel Prize in 1969 for this work, grew individual populations of the same bacteria in parallel cultures and exposed these cultures to a bacteriophage. They then counted the number of surviving bacteria – those that had acquired a mutation that enabled them to resist the virus – in each parallel culture. Some cultures exhibited a relatively large number of survivors, while other cultures showed very few, if any survivors with many between these extremes. This “fluctuation” was the result of random mutations at different times during the growth of the bacteria prior to exposure to the virus. If a mutation that conferred resistance to the virus occurred early in the growth of a particular culture, there was more time for that variant to reproduce, thus generating larger numbers of resistant cells. By contrast, if a random mutation that conferred resistance to the virus occurred late in the growth of a particular culture, there was little time for that variant to reproduce, generating fewer resistant progeny cells. Exposure of cells to the virus clearly did not induce mutations to resistance; it only selected for pre‐existing mutants that conferred resistance.

Patients who died because of lack of healthcare will not be counted in the statistics of COVID‐19 deaths but are nonetheless casualties of the pandemic.

Similarly, infection of a vaccinated individual with SARS‐CoV‐2 does not induce mutations in the viral genome to resist or evade immunity. However, if a pre‐existing variant exhibits resistance to immunity, that variant will be capable of growing in the vaccinated host. This is the basis of the breakthrough infections we are witnessing with the latest variants.

## Vaccines still confer at least some protection against all variants

This is not the only pseudoscientific argument grounded in a wilfully wrong interpretation of evolutionary biology. Another objection to vaccination from one of the emails I received, is “the virus has mutated to a point where the vaccines do not protect”. This is again false, at least for the variants that currently circulate among the human population. Vaccinated people show a much milder disease progression, have very low rates of hospitalization and even lower mortality (Klompas, 2021). There are studies showing that the viral load of the Delta variant in breakthrough infections in the vaccinated is only high at the onset of the disease but is quickly brought under control (Singanayagam *et al*, 2022), while the unvaccinated continue to grow enormous amounts of virus and it is mostly the unvaccinated who are populating hospitals and morgues. One reason why the vaccines still confer protection despite the new mutations of the virus is that the human receptor for spike protein (angiotensin‐converting enzyme 2, ACE2) is fixed, which puts a limitation on how mutable the spike protein can get and still attach to the host receptor.

The unvaccinated are also forcing rationing of health care, filling hospital beds to the extent that patients in need of treatment for other life‐threatening conditions have experienced difficulties in obtaining care (https://www.nytimes.com/2021/12/08/opinion/covid‐michigan‐surge.html). Patients who died because of lack of healthcare will not be counted in the statistics of COVID‐19 deaths but are nonetheless casualties of the pandemic.

More misinformation from another email: “This virus is arguably the most infectious virus in history that mutates incredibly fast”. As viruses go, SARS‐CoV‐2 does not mutate incredibly fast, because its RNA polymerase possesses an error‐correcting mechanism (Robson *et al*, 2020). The rate of mutation is nowhere near that of viruses such as HIV (Roberts *et al*, 1988). The reason we are seeing a lot of variants of SARS‐CoV‐2 is simply the sheer numbers of virus growing in the unvaccinated. For example, compare an error rate of 10 per 100 copies to 1 per 100 copies. If the 10% error‐rate virus makes 10,000 copies, there will be 1,000 mutants. But if the 1% error‐rate virus makes 1,000,000 copies, there will be 10,000 variants.

Another correspondent wrote “It is obvious the vaccines have done nothing to slow COVID‐19”. It is not that the vaccines have failed to slow COVID‐19: it is the unvaccinated who have failed to slow COVID‐19. The Omicron variant was first reported in South Africa, which at the time had a vaccination rate of about 30% (https://www.nytimes.com/interactive/2021/world/covid‐vaccinations‐tracker.html). In a partially vaccinated population, the virus has plenty of opportunity to generate variants in the unvaccinated, and the variants have plenty of opportunity to test their fitness among the vaccinated. If and when a variant exhibits complete resistance to the vaccine, then all those presently vaccinated will rejoin the unvaccinated as breeding grounds for still more variants, and new vaccines will become necessary. It is therefore very much in the self‐interest of wealthy nations with access to vaccine supplies to facilitate extensive vaccination in poorer countries. The longer societies sustain a huge unvaccinated population, the greater the chances of a doomsday variant emerging.

The longer societies sustain a huge unvaccinated population, the greater the chances of a doomsday variant emerging.

Other objections relate to the relatively lower mortality of SARS‐CoV‐2 compared to the 2003 SARS‐CoV‐1 virus: “This isn’t the extremely deadly virus you think it is, and we have drugs that can treat it. So just remember that governments are locking down unvaccinated people and putting people in quarantine camps for a virus with a death rate of 1%”. SARS‐CoV‐1 had a death rate of ~ 10% (Chan‐Yeung & Xu, 2003) and we do not know the real death rate of SARS‐CoV‐2 because of vaccination. In addition, many survivors suffer from serious long‐term conditions. Generally, society makes a judgement about what death rate is acceptable versus the benefits and/or efforts to prevent it. Every year, people die in plane crashes, but the low death rate is accepted because of the benefits of flying. One can make the case that a 1% death rate – if it is that low – should be acceptable; personally I think it is too high given that vulnerable groups have a much higher risk of death and disability.

## Other arguments against vaccination are also specious

Some remain unvaccinated not for ideological reasons but because they are suspicious of the mRNA technology and are waiting until protein‐based or attenuated‐virus vaccines become available. While their hesitancy is somewhat understandable, it is nevertheless a danger to society. So far, mRNA vaccines have been shown to be at least as safe as vaccines based on viral proteins or attenuated‐viruses. There may be uncertainty about potential long‐term effects caused by the chemical modification of the RNA to increase its half‐life in the target cell (although this modification is itself a natural constituent of all cells) or the lipid carrier to deliver the RNA but billions of doses have now been administered worldwide with rare reports of serious adverse reactions (https://www.cdc.gov/coronavirus/2019‐ncov/vaccines/safety/adverse‐events.html).

Other anti‐vaccination rhetoric compares vaccine and quarantine mandates to totalitarian states, including Nazi Germany. One correspondent entreated me to stop “turning people against each other and instead encourage people's freedom to choose”. But if the “freedom to choose” results in the death of others, what kind of freedom is that? Freedom to kill? A subtle variation of this argument was published in a letter to *Lancet*, in which the author argues that “[h]istorically, both the USA and Germany have engendered negative experiences by stigmatising parts of the population for their skin colour or religion” (Kampf, 2021). This is a false analogy. The unvaccinated are not a fixed group: anyone can leave it instantly by getting vaccinated. It is not the same thing as bigotry based on racial, ethnic or religious grounds. The choice whether to vaccinate or not is therefore not just about an individual’s freedom – it is about accepting and abiding by the rules of society, the “social contract”.

I do believe that medical exemptions are legitimate. For example, a relative of mine has an aortic aneurysm, and stress of vaccination could increase the risk of bursting that aneurysm, which would almost certainly be fatal. However, arguments such that nursing mothers, or prospective mothers trying to conceive should be medically exempt do not hold up to scrutiny, as there is evidence that vaccinations do not interfere with these pursuits (https://www.cdc.gov/coronavirus/2019‐ncov/vaccines/recommendations/pregnancy.html) and if anything, a vaccinated nursing mother would pass protective immunity to her infant. Religious exemptions, on the other hand, are rarely, if ever justifiable. Indeed, with few exceptions, the world’s major organized religions either support or at least do not oppose vaccination (https://www.vumc.org/health‐wellness/news‐resource‐articles/immunizations‐and‐religion; https://www.cnn.com/2021/09/09/health/covid‐vaccine‐religious‐exemptions‐khn/index.html).

Had the Internet existed in 1950, we would still be suffering from smallpox and polio, as misinformation about vaccination is too easily amplified over social networks and with far greater reach. There is some sense of deja vu here as similar objections were raised when the MMR (Measles, Mumps, Rubella) vaccine was introduced. The infodemic – the rapid spread of misinformation and propaganda – may therefore require similar “hygiene measures” and “vaccination” in the form of better education, which “in the long run, may contribute to a society that is more immune to infodemics” (Niemiec, 2020).

Had the Internet existed in 1950, we would still be suffering from smallpox and polio, as misinformation about vaccination is too easily amplified over social networks and with far greater reach.

Universal vaccination, with a few medical exemptions, would help to stop or slow the generation of new variants. Growth of virus in the unvaccinated is orders of magnitude greater than growth in breakthrough infections of vaccinated people. We are all in this together, and the pseudoscience encouraging anti‐vaccination rhetoric is only prolonging the pandemic and might generate even more dangerous variants of the virus.

## Supporting information



Review Process File
