# Supplementary material for: Lamarck redux and other false arguments against SARS‐CoV‐2 vaccination: Pseudoscientific arguments against vaccination do not stand up to scrutiny
Source: EMBO Rep. 2022 Feb 23;23(4):e54675. doi: 10.15252/embr.202254675 (PMC8982595; doi:10.15252/embr.202254675)
Supplement: Supplementary file 1 — Review Process File [file EMBR-23-e54675-s001.pdf]

EMBO reports

Lamarck redux and other false arguments of SARS-CoV-2 vaccination

Emanuel Goldman
DOI: 10.15252/embr.202254675

Corresponding author(s): Emanuel Goldman (egoldman@njms.rutgers.edu)

**Review Timeline:**	Submission Date:	17th Jan 22
	Accepted:	10th Feb 22

Editor: Holger Breithaupt

Transaction Report:

This article was editorially reviewed.”

(Note: With the exception of the correction of typographical or spelling errors that could be a source of ambiguity, letters and reports are not edited. Depending on transfer agreements, referee reports obtained elsewhere may or may not be included in this compilation. Referee reports are anonymous unless the Referee chooses to sign their reports.)
